# Reliability for music-induced heart rate synchronization

**DOI:** 10.1038/s41598-024-62994-0

**Published:** 2024-05-28

**Authors:** Ryota Nomura

**Affiliations:** Waseda University, 2–579–15, Mikashima, Tokorozawa, Saitama Japan

**Keywords:** Human behaviour, Circulation

## Abstract

Common inputs synchronize various biological systems, including human physical and cognitive processes. This mechanism potentially explains collective human emotions in theater as unintentional behavioral synchronization. However, the inter-subject correlation of physiological signals among individuals is small. Based on findings on the common-input synchronization of nonlinear systems, we hypothesized that individual differences in perceptual and cognitive systems reduce the reliability of physiological responses to aesthetic stimuli and, thus, disturb synchronization. We tested this by comparing the inter- and intra-subject Pearson’s correlation coefficients and nonlinear phase synchronization, calculated using instantaneous heart rate data measured while appreciating music. The results demonstrated that inter-subject correlations were consistently lower than intra-subject correlations, regardless of participants’ music preferences and daily moods. Further, music-induced heart rate synchronization depends on the reliability of physiological responses to musical pieces rather than mood or motivation. This study lays the foundation for future empirical research on collective emotions in theater.

## Introduction

Music has touched the hearts of people from ancient times to the present in most cultures. The ubiquity of music in human society has led many to take it for granted, and few have questioned its ability to evoke human emotions. Critical music aesthetics is a legitimate discipline, yet it has often focused on the individual process of an audience’s aesthetic experience. Therefore, the mechanisms that evoke similar feelings among audience members have remained unclear.

Common input synchronization could explain how music induces collective emotional responses. It is a phenomenon where systems receive an identical input and align their output sequences. In various situations, a common input can align output oscillators and often provides a large coherent output among systems. Thus, the common input synchronization plays a crucial role in collective behavior in many situations as a universal mechanism. A variety of inputs, such as periodic, pulse^1^ and noise^2^ inputs, can synchronize uncoupled systems. Therefore, common-input synchronization appears in many fields, including physics^3,4^, optics^5^, biology^6,7^, and psychology^8,9^.

Regarding biological organisms, common-input synchronization has been widely studied across different spatial scales, from the macroscale synchronization of populations of two isolated islands by climatic variations^7^ to the microscale reproductive firing timing of a single neuron across trials^6^. Regardless of apparent differences, these phenomena can be discussed in terms of consistency. Consistency is defined^5^ as the reproducibility of outputs among nonlinear systems, with arbitrary parameters and different initial states, by repeatedly applying an identical input. A single neuron, for example, reproduces millisecond reliable firings from receiving a frozen noise, whereas initial states vary across trials^6^.

Since the mechanism of synchronization by common input is broadly applicable, we predict that biological rhythms synchronize with each other through music listening. In other words, commonly applied music acts on the information processing systems of individuals in different initial states, causing similar output as biological oscillators. People are nonlinear systems that respond to input music. From the viewpoint of consistency, therefore, synchronization is induced by an identical input applied to different individuals, and that repeatedly applying the input to the same individuals results in an essentially equivalent phenomenon—synchronization induced by a common input which is applied to nonlinear systems with different parameters of internal noise and initial states. In both cases, we can consider it as music-induced heart rate synchronization from the viewpoint of consistency.

Recent studies have shown that the physiological signals of different individuals can be synchronized even in the absence of direct coupling. For example, identical narratives^8,9^ significantly correlate the heart rates of individual participants. Researchers have suggested that the inter-subject correlation (ISC) depends on how similarly the participants consciously process the given information. As the participants’ brains and heart rates are correlated, a common audiovisual stimulus can synchronize the heart rates of different individuals if these individuals consciously process this information in similar ways. This mechanism has been supported by a lack of significant ISC in participants with impaired consciousness and predictability of memory performance by the extent of ISC^9^.

However, ISCs calculated using physiological signals, including heart rate, are usually low (*r* $\approx$0.10)^8,9^ compared to correlations calculated using the signals across their modalities within individuals ($r\approx$ 0.60)^9^. Previous studies^8,9^ focusing on the synchronization induced by an identical input applied to different individuals suggest that the low correlation is due to weak intra-person correlation between the conscious processing and heart rate. This intra-person correlation is physiologically constrained and, thus, is challenging to control directly. In this study, instead, we controlled the degree of noise by eliminating individual differences in the conscious processing by repeatedly applying identical input to the same individuals. Theoretical and empirical studies^2,5,6^ on dynamical systems suggest that synchronization can be achieved when the internal noise of the target systems is small relative to the input, and when the input amplitude is small enough not to destabilize the system dynamics. In the case of music appreciation, lower noise that increases the reliability of physiological responses to the identical stimulus is expected to enhance correlation. A study using spontaneous blinks^10^, which partially depend on information processing, also suggests that reduction of internal noise enhances synchronization. During performance appreciation, spontaneous blinking of participants with more viewing experience was more synchronized than that of participants with less viewing experience. Participants’ experiences guide their information processing and, thus, decrease internal noise while response blinking to the performance video. These findings support the hypothesis that enhanced reliability increases synchronization.

To directly examine this hypothesis, we controlled the reliability of physiological response to aesthetic input by focusing on the variability between and within participants. First, we assumed that individual cognitive characteristics remain stable across different days. In other words, parameters of the perceptual and cognitive systems are essentially the same within the target individual. Meanwhile, individuals’ moods fluctuate each day. We, thus, assumed that daily moods can act as initial states while they are the appreciating music at each trial.

**Figure 1:**
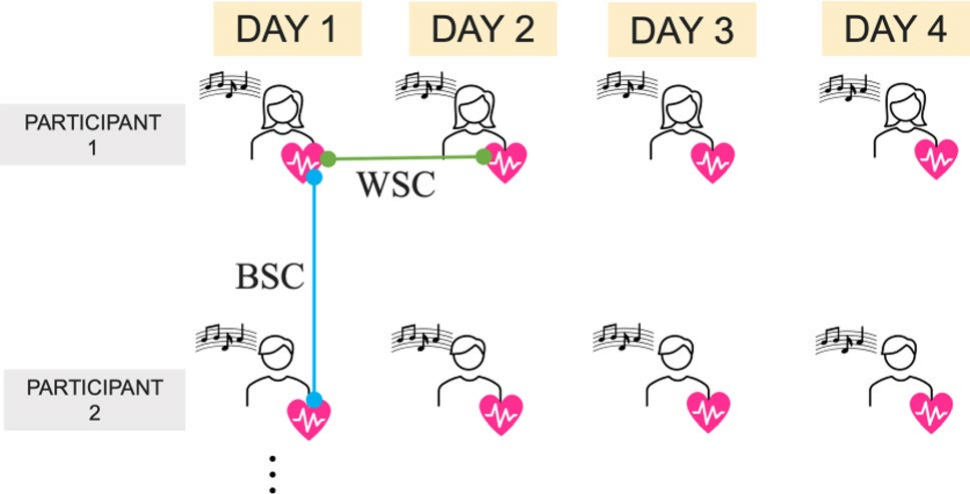
Trials for repeated measurement. All participants completed the experiment four times at 2- to 7-day intervals. The green link shows the calculation of the within-subject correlation (WSC), and the blue link shows the calculation of the between-subject correlation (BSC).

Here, we examined whether synchronization depends on the reliability of physiological responses to musical pieces under the assumption that intra-person diurnal variations are smaller than inter-person differences. Specifically, we conducted a psychophysiological experiment in which participants were randomly presented with pieces of music while their heart rates were measured. As shown in Fig. 1, we compared the correlation coefficients of the heart rates of participants independently listening to identical music (between-subject correlation [BSC]) and the correlation coefficients of the heart rates of participants listening repeatedly to a participant (within-subject correlation [WSC]).

**Figure 2:**
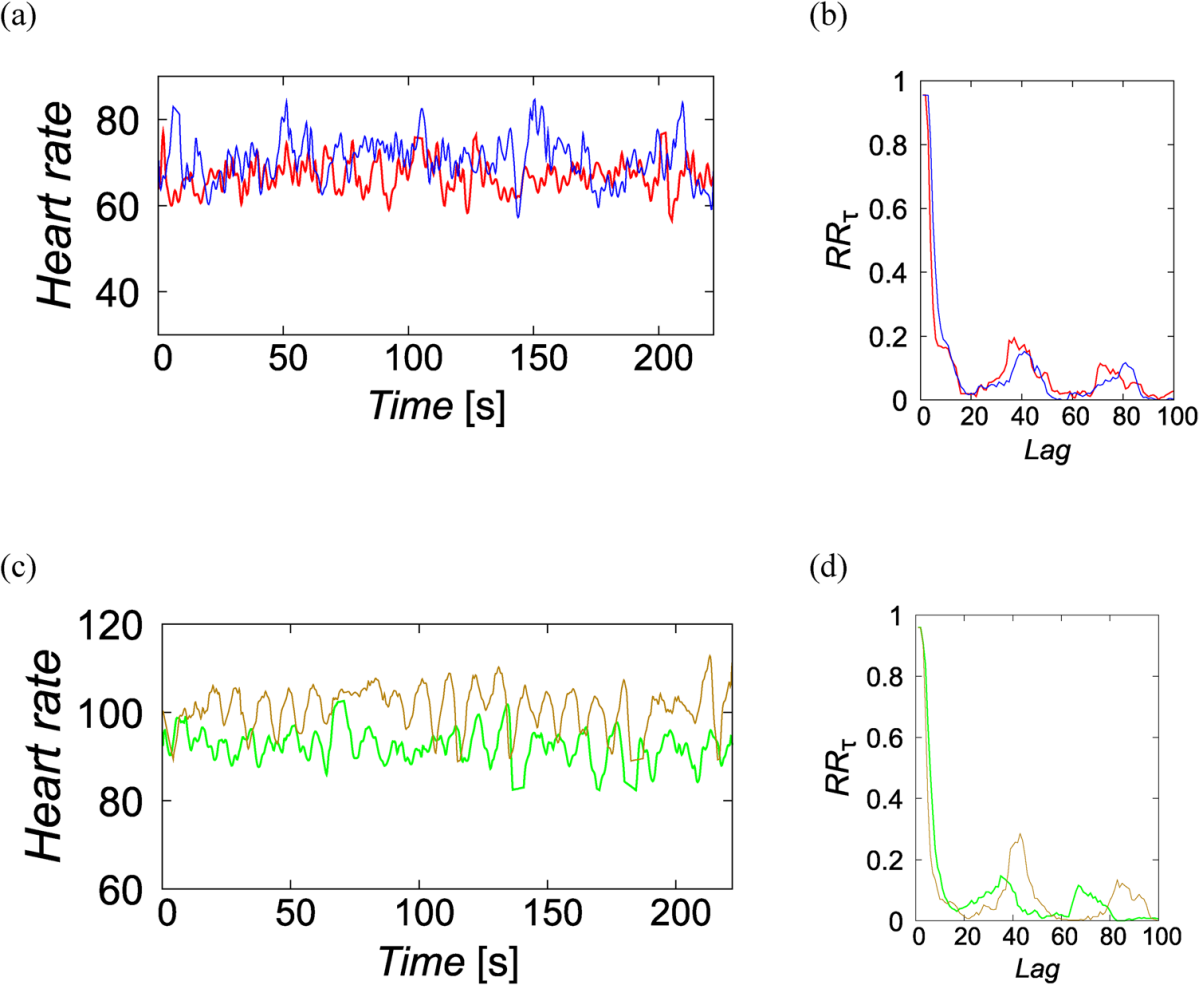
Examples of phase synchronization. (**a**), (**c**) Pairs of instantaneous heart rates and (**b**), (**d**) recurrence rate at each lag $\tau$ when phase synchronization is high ($\varphi = 0.79$) and low ($\varphi = 0.27$), respectively. Although the first-order correlations of instantaneous heart rates in (a) and (c) are at almost same level, $r=0.07$ and $r=-0.08$, the recurrence rates better coincided in (**b**) than that of (**d**).

In the previous study^9^, the first-order correlation was used and, therefore, nonlinear synchronization is still unclear. If the music is influential enough, the state of the heart rate dynamics would converge into a similar state through music appreciation, regardless of the participants’ initial states. In this case, nonlinear synchronization can quantify the relationship between heart rates by considering the dynamics of the complex system in a state space^11^. Hence, the index of nonlinear synchronization can be higher even if the linear correlation coefficient is low (Fig. 2). Thus, we calculated phase synchronization that quantifies the coincidence of recurrence in the state space by following Takens’s embedding theorem^11^ of dynamical systems (for details, see the Methods section). Furthermore, we also compared the between-subject nonlinear phase synchronization, between-subject synchronization [BSS], and the within-subject nonlinear phase synchronization, within-subject synchronization [WSS].

To exclude the effect of music preference, we used both a piece of music selected by the experimenter (referred to in this paper as “the prepared music”) and a piece of music selected by the participant, which the participant had previously been very moved by (“the favored music”). We made the following natural assumptions regarding music appreciation: First, there exist positive correlations when the valence of the moods coincides (between positive moods and between negative moods) and negative correlations when they do not (between the positive and the negative moods). Additionally, participants were consistently more moved by the favored piece than by the prepared piece, regardless of repetitions. Furthermore, the positive mood experienced prior to listening to the musical piece positively correlated with the extent of the felt moved to the piece, but the moods also exhibited positive changes after listening to the pieces.

Based on these assumptions, subjective responses regarding daily moods and the emotions associated with being moved by the music were also considered as potential variables for synchronization. The trial was repeated four times at intervals of a few days to obtain the BSC, WSC, BSS, and WSS of the participants’ instantaneous heart rates. Changes in mood on the same day were also assessed by asking the participants to respond to a state-emotion scale. Notably daily mood was not manipulated as an independent variable. Rather, we examined whether the similarity of the mood as an initial state that potentially predicts the correlations between heart rates.

Results showed that WSCs (both for the prepared and favored pieces) were consistently higher than BSC, indicating that the reliability hypothesis is supported. Nonlinear analysis also demonstrated the same results: WSSs (both for the prepared and favored pieces) were higher than BSS. The moods ratings had almost no predictive power for WSC and WSS, indicating diurnal variations of moods cannot explain the heartrate correlation, which has temporal dynamics. All results supported the reliability hypothesis.

## Results

### Internal consistency of the mood subscales and relationship between mood and music appreciation

First, to examine the statistical reliability of the scales, alpha coefficients were calculated for the state-emotion scale that measures moods using eight subscales. The coefficients of the subscales were $\alpha \text{s}\approx .80$, except for the subscale of hostility, measured prior to music appreciation, which was $\alpha = .67$. The alpha coefficients of the scale measuring the degree to which a participant was moved by the music were also sufficiently large ($\alpha = .83$ for prepared piece and $\alpha = .80$ for favored piece). As we found basically sufficient internal consistency in these subscales, the mean value for each subscale was used.

Then, to examine the effects of music type (prepared or favored) and repetition of appreciation (1^st^—4^th^ trials), we performed a 2 × 4 ANOVA using the score of the degree to which a participant was moved by the music as a dependent variable. As predicted, music type had a main effect ($F(\text{1,128})= 4.961,p=.028$), indicating that the scores for the favored pieces were consistently higher than that for the prepared piece. Other main effect and interaction were not significant.

Next, to detect interrelationships among the moods, correlations between subscale scores were calculated using data from all trials for both prepared and favored pieces. Almost all correlation coefficients were significant (Table 1). To determine the relationship between these moods and the extent of being moved, we also calculated correlation coefficients between them. The results for the prepared piece showed that boredom ($r= -.389, p<.01$), active pleasure ($r= .560, p<.01$), politeness ($r= .578, p<.01$), concentration ($r= .357, p<.01$), and surprise ($r= .355, p<.01$) correlated with the extent to which the participants were moved. For favored pieces, boredom ($r= -.252, p<.05$), active pleasure ($r= .470, p<.01$), and politeness ($r= .349, p<.01$) correlated with the extent to which the participants were moved but not with concentration or surprise. These results indicate that the listener’s pre-appreciation mood is related to how much they would be moved by a piece of music.

**Table 1:** Correlation coefficients between mood variables.

	1	2	3	4	5	6	7	8
1. Depression /Anxiety	–	.441**	.540**	− .214*	− .123	− .090	.395**	.386**
2. Hostility		–	.324**	− .094	.023	.094	.286**	.354**
3. Boredom			–	− .465**	.029	− .370**	.131	.033
4. Active pleasure				–	.238**	.637**	.252**	.198*
5. Passive pleasure					–	.352**	.316**	-.093
6. Politeness						–	.388**	.271**
7. Concentration							–	.234**
8. Surprise								–

*N* = 136, *p < .05, **p < .01.

Finally, we examined the variability of mood by music appreciation. A 2 (pre- and post-appreciation) × 2 (music type) MANOVA revealed that the scores for boredom ($F(1 ,134)= 4.149,p=.044$), active pleasure ($F(\text{1,134})= 4.238,p=.041$), and politeness ($F(\text{1,134})= 9.910,p=.0002$) scales varied before and after appreciation; The boredom score decreased and the active pleasure and politeness scores increased. However, differences of the scores were smaller from 0.200 to 0.365. No other variables were statistically significant. Overall, the assumptions regarding music appreciation were confirmed.

### Correlation coefficients were on average higher within subjects than between subjects for both the prepared and favored pieces

**Figure 3:**
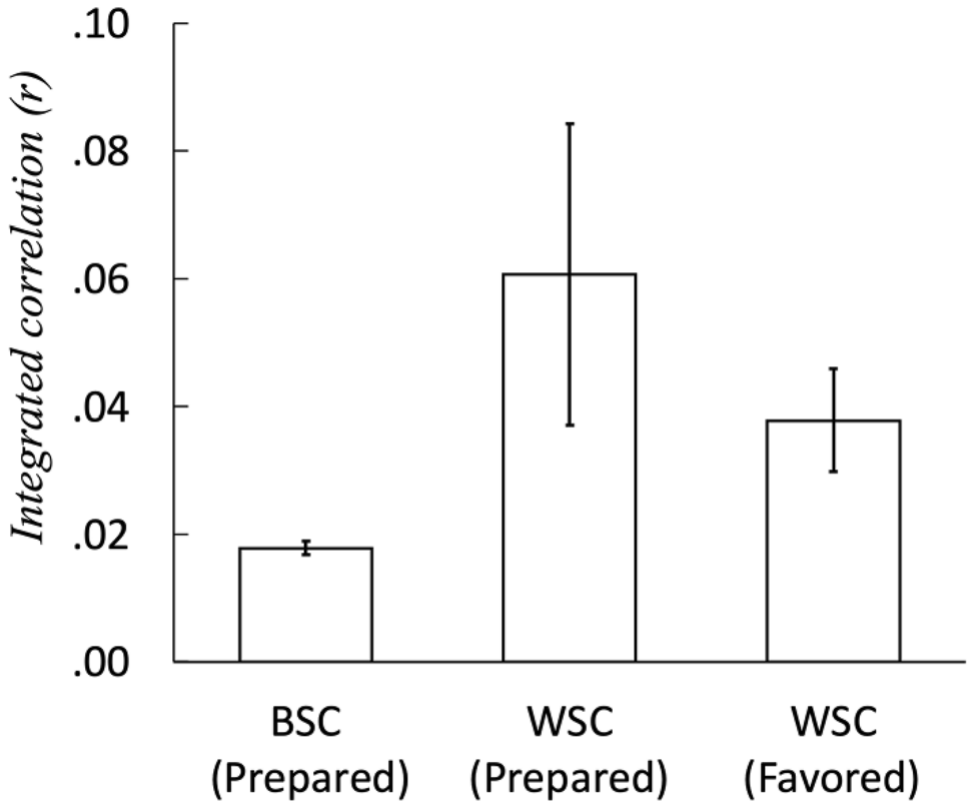
The integrated correlation coefficients. Correlations for each participant integrated with the meta-analytic method. BSC: between-subject correlation; WSC: within-subject correlation. The error bars show the standard deviations.

We calculated the BSC using four randomly selected instantaneous heart rates obtained from different individuals listening to the prepared piece. The 1,000 times simulations showed *r* = 0.0178 [CI:0.0157–0.0199]; therefore, we rejected the null hypothesis that the correlation coefficient equals zero (Fig. 3, BSC). This value was approximately the same level as the BSC calculated using all the pairs of the instantaneous heart rates of the 17 individuals (_17_C_2_ = 136) in each trial (not shown in Fig. 3). For the 1st trial, *r* = 0.0283 [95%CI 0.0227–0.0340]; for the 2nd trial, *r* = 0.0171 [95%CI 0.0114–0.0227], for the 3rd trial, *r* = 0.0006 [95%CI − 0.0050–0.0063 ]; and for the 4th trial, *r* = 0.0121 [95%CI 0.0066–0.01719]. Overall, *r* = 0.0146 [95%CI − 0.0184–0.0475]. In most cases, the null hypothesis that the correlation coefficient is zero was rejected. Therefore, the music-induced heart rate synchronization among individuals was found as BSC, although this value was slightly smaller than that for the ISC (r = 0.04 [95%CI: − 0.01–0.10]) reported in a previous study^9^ discussed above in which a narrative was used as a common input. Note that random combinations could not be computed for the favored pieces of music because the pieces differed among the participants.

The WSC calculated using data on the participants’ instantaneous heart rates during the prepared piece across the four trials was *r* = 0.0607 [95%CI 0.0146 – 0.1069] (Fig. 3, WSC [Prepared]). Similarly, the WSC calculated using data on the participants’ instantaneous heart rates during the favored piece across the four trials was also significantly higher than zero, at *r* = 0.0378 [CI:0.0220–0.0535] (Fig. 3, WSC [Favored]). Both types of WSC were significantly higher than BSC. The differences WSC for the prepared and favored pieces was not significant.

### Phase synchronization coefficients were on average higher within subjects than between subjects for both the prepared and favored pieces

**Figure 4:**
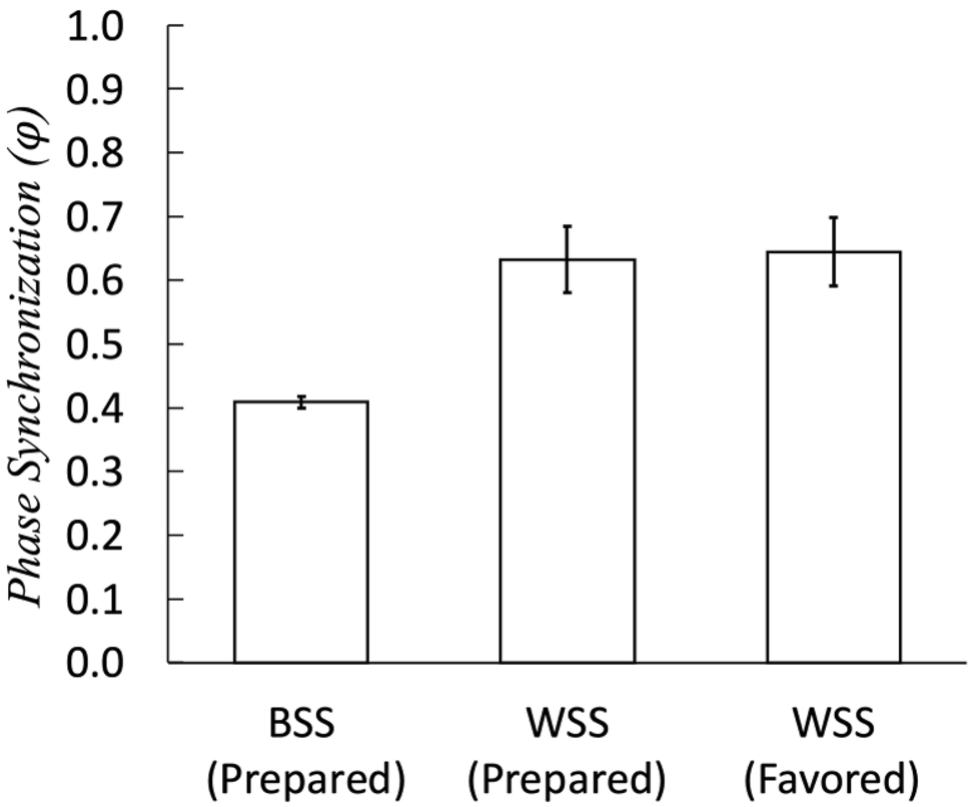
The phase synchronization. BSS: between-subject synchronization; WSS: within-subject synchronization. The error bars show the standard deviations.

To identify generalized synchronizations that do not always appear as linear correlations, we also calculated nonlinear correlations. Based on the recurrence plots, we could detect the synchronization between two time series using the phase synchronization coefficient. Recurrence plots are used to identify linearity, stability, determinism, and other properties of a system based on dynamical systems theory. The phase synchronization ($\varphi )$ quantifies the degree of coincidence between recurrence probabilities that correspond to recurrences in the state space, represented as recurrence plots (Fig. 2). This is normalized to be from zero (no coincidence) to unity (perfect coincidence). For BSS, the 1,000 times simulations showed $\varphi = 0.409$[CI: 0.3994–0.4180] (Fig. 4). The WSS calculated using the four trials for each participant were $\varphi = 0.633$ [95%CI 0.580–0.684] (Fig. 4, WSS [Prepared]) and $\varphi = 0.645$ [95%CI 0.5907–0.698] (Fig. 4, WSS [Favored]). Intra-individual WSSs consistently showed higher phase synchronization than inter-individual BSS.

### Prediction of WSC based on mood variations

As suggested in the Introduction, music-induced heart rate synchronization may also be influenced by motivational variables other than the system reliability. Thus, we first examined whether pre-appreciation moods correlated with the degree to which participants were moved by listening to music. To detect whether intra-individual diurnal variability could explain heart rate synchronization, we examined the effect of pre-appreciation mood on WSC. The results demonstrated that none of the moods predicted the degree of Pearson correlation based WSC during the appreciation of the prepared piece (${R}^{2}\text{s}< 0.004, ps>.10$). When phase synchronization WSS was predicted by the same independent variables, only active pleasure was significant ($t= -0.200, p=.048$). In both cases, for WSC and WSS, mood did little to predict the degree of synchronization.

## Discussion

In this study we introduced music-induced heart rate synchronization as a common-input synchronization of human unintentional behavior. By controlling the individual differences, we examined the reliability hypothesis. The results of the psychophysiological experiment showed that the intra-individual WSCs were higher than the inter-individual BSC. The WSCs were approximately at the same level, compared to the previously reported ISC^9^. Similarly, for nonlinear synchronization, intra-individual WSSs were higher than inter-individual BSS. The differences between intra-individual indices for the favored pieces and the prepared pieces were not significant.

The synchronization within individuals was stronger than the synchronization between individuals, indicating that smaller differences in parameters contribute to synchronization. Moreover, the preference of musical piece had no effect. Theoretical studies have shown that common input synchronization of systems with different initial values requires that the internal noise be small^5,6^. Given that the input is constant across trials, these results support the reliability hypothesis that the input–output reliability of a participant’s perceptual-cognitive system enhances music-induced heart rate synchronization under the realistic assumption that intra-individual diurnal variability is smaller than individual differences. A previous study^12^ reported decreased ISC when using electroencephalograms (EEG) and music pieces presented in immediate succession. In this study, however, the effect of decreased synchronization was not demonstrated, suggesting that the effect of repetition does not persist for several days.

Moods were correlated with each other and with the degree to which a participant was moved by the music. Moods varied positively through music appreciation: Decreased boredom, increased active pleasure, and increased politeness. At the same time, these moods of boredom, active pleasure and politeness predicted the extent of being moved by the prepared and favored pieces. Nonetheless, these moods did not predict WSC. Regarding WSS as well, only the mood of active pleasure predicted phase synchronization and the others did not. Although moods as an initial state before listening to music leads to feel moved and, in turn, is enhanced through music appreciation, daily moods were found to have little effect on synchronization. Music-induced synchronization of brain activity measured using an EEG^13^ suggests that ISC depends on responses to localized musical components, such as rhythms and melodies, rather than individual musical preferences. Therefore, for synchronization to occur, the heart rate must fluctuate in response to the structure of the music as an input. However, as a diurnal variation, mood does not have these short-term (i.e., seconds- and minutes-long) temporal dynamics. Thus, diurnal variations would not contribute to music-induced heart rate synchronization. In addition to the direct effects of mood, there is also the possibility that mood influences attention, which in turn may modulate the degree of synchronization. Future studies could consider this perspective by measuring attentional indices.

The heart rate varies with an autonomous cycle and the time series usually exhibits a relatively slow approximately 5-s period. The primary role of the heartbeat is blood circulation, and the changes are not so rapid under the circumstance of music listening. Therefore, the heart rate correlation would not correspond one-to-one to the structure of the music although EEGs have been shown to be influenced by the higher-order structure of music^13^. Simultaneously, this consideration suggests that collective information processing^9^ increases the reliability of physiological responses to an input. This reliability hypothesis provides a new perspective for enhancing music-induced heart rate synchronization. One possible application is the use of a device that provides tactile stimuli accompanying music; if the stimuli increase the reliability of the system, then this method may enhance the music-induced heart rate synchronization.

This study had several limitations. First, the piece of music used in the experiment was confined. As synchronization was detected in conditions of favored pieces, we can assume that music generally synchronizes heart rates among people, including in the cases of repetitive presentations to the same individual. However, it is unclear how characteristics such as the appeal of a musical piece or its popularity^14^ affect this synchronization. Second, this study did not examine which musical elements contribute to synchronization. Music-induced heart rate synchronization potentially induces a strong coherent output behavior in daily lives of individuals as well as at music concerts^15^. Elucidating the specific components of the aesthetic appeal of popular music would contribute towards the creation of affective musical pieces.

Overall, this study showed that music-induced heart rate synchronization—a form of unintentional collective human behavior—is realized by the reliability of the physiological response to the music input. From the perspective of consistency, intra-individual synchronization and inter-individual synchronization can be explained from the same framework. This can be described as a matter of degree rather than an absolute difference. This brings us to the next research question: Does similarity in terms of specific cognitive or personality traits promote synchronization among individuals? The tendency toward synchronization of heart rate and psychological similarity among audience members may contribute to the excitement that occurs at live music concerts where only fans are present, compared to the emotional responses of a variety of members. This suggests that common-input synchronization may be the mechanism underlying the temporally coherent behavior of a large audience in a theater. However, it should be noted that studies conducted in theaters^16–18^ tend to demonstrate higher correlations than other experiments conducted with individuals in laboratories^8,9^. In a theater, therefore, audience interactions^19^ may enhance music-induced synchronization. Future research should examine whether unconscious social interactions among audience members contribute to synchronization by reducing differences in responses to input performance. Meanwhile, the present study contributes to empirical research on collective behavior among audience members in a theater as a mesoscale biological phenomenon.

## Methods

### Participants

Seventeen Japanese university students (11 males and six females) participated in this study. Although the participants all enjoyed listening to music on a daily basis, none had a specialized education in music. The mean age of the participants was 22.17 years (SD = 0.63). The sample size was 68 because each participant attended four trials. To participate in the study, the students had to (a) already have a subscription to a music application, (b) have access to the prepared and favored piece of music, (c) have no problems listening to music using earphones, and (d) have no health problems or psychological resistance to wearing the electrode pads used to measure heart rate.

### Pieces of music

We selected a prepared piece of music for all participants to listen to and asked the participants to choose their favorite piece of music to listen to. The participants accessed the pieces through music subscription services for which they paid monthly fees. We chose the Japanese version of *For the First Time in Forever* (Disney) as the prepared piece. The song was 222 [s]. The piece was selected because it is well-known among Japanese graduate students and is considered to have appeal.

For the favored pieces of music, we asked the participants to choose the most moving piece of music they had ever listened to. Most pieces were performed by Japanese artists and were in the genres of pop and rock. The mean length of the pieces was 227.88 [s] (SD = 51.04 [s]; range: 154 [s] to 363 [s]). Before participating in the experiment, the participants completed an online questionnaire. The survey asked them to list the title of their favored piece and the lyrics or performance techniques they found particularly moving. Each participant used the same favored piece across all trials.

### Subjective ratings

The questionnaire contained two scales. The first scale included items regarding emotional states/daily moods and the second scale included items about the experience of being moved by a piece of music. The first multidimensional state-emotion scale^20^ included depression/anxiety, hostility, boredom, active pleasure, passive pleasure, politeness, concentration, and surprise. These eight subscales were measured using shortened versions with five items each. To respond to the items, the participants used a five-point scale ranging from “not feeling at all” (0) to “feeling clearly” (4).

The second scale contained 12 items that measured the emotion of being moved while appreciating music^21^. The following phrases describing the emotion of being moved along with the values or concepts related to it were used: full of heart (love, good, tears), joyful (happiness, shouting with exultation), and awe (surprising, speedy, and big). Participants responded using a five-point scale ranging from “not feeling at all” (0) to “feeling clearly” (4).

### Heart rate measurement

Participants’ heart rates were measured using a wearable device (MyBeat, WHS-3). An electrode pad was placed under each participant’s collarbone, approximately 3 [cm] above the epigastrium to which the wearable device was attached. The device read the heartbeat waveform and recorded the time at which the R-wave occurred. Data were saved on an iPhone. Music was played at preset times during each trial to coincide with the start of the measurement.

### Procedure

**Figure 5:**
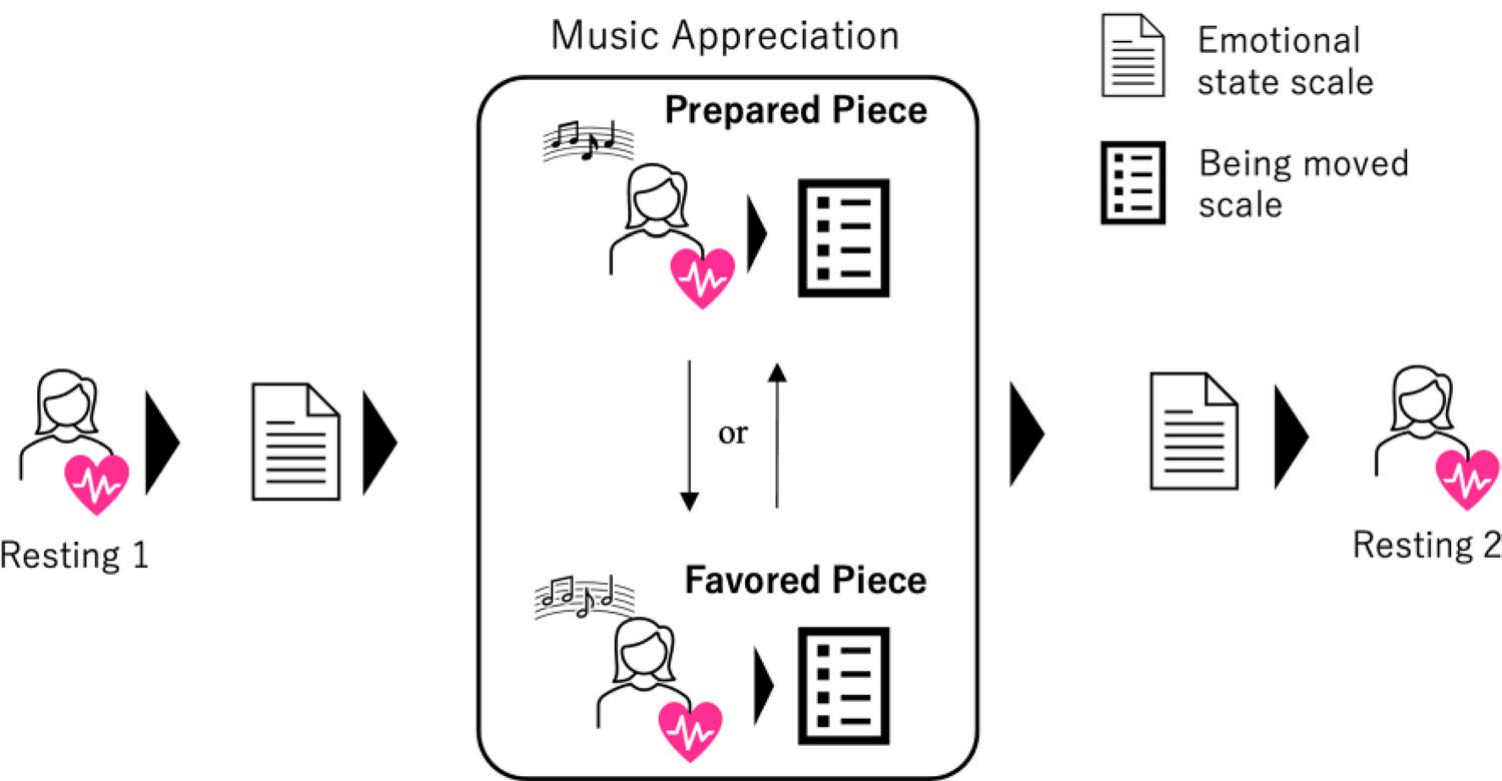
Procedure of the experiment. The orders in which the participants listened to the prepared and favored pieces of music were randomly assigned for each trial.

First, the experimenter asked the participants to enter the room and instructed them to wear an electrode pad with a wireless device to measure their heart rates. The experimenter then tested whether the heart rates were measured correctly; if no problems were observed, resting heart rate intervals were measured over a period of 240 [s]. To eliminate the influence of visual information, the participants were asked to close their eyes during the heart rate measurements. After the resting measurement, the participants completed the state-emotion scale^20^ to assess their mood. The participants listened to the favored and prepared pieces sequentially while their heart rates were measured. The order in which the participants first listened to the pieces was counterbalanced (Fig. 5). Under both conditions, all participants closed their eyes while appreciating the music.

The participants then completed the state-emotion scale again. The participants’ resting heart rate intervals were measured again over a period of 240 [s] while their eyes were closed. In the first trial, the participants answered a questionnaire to identify their daily experiences as listeners or music performers. This trial was repeated four times per participant, with periods ranging from two to seven days (Fig. 4).

All procedures of the experiment in this study were approved by the Ethics Review Committee on Research with Human Subjects of Waseda University (“Research on physiological and subjective responses during music appreciation,” No. 2021–013). Written informed consent was obtained from all participants included in this study. All research was performed in accordance with the Declaration of Helsinki and the relevant guidelines in Japan.

### Analysis

**Figure 6:**
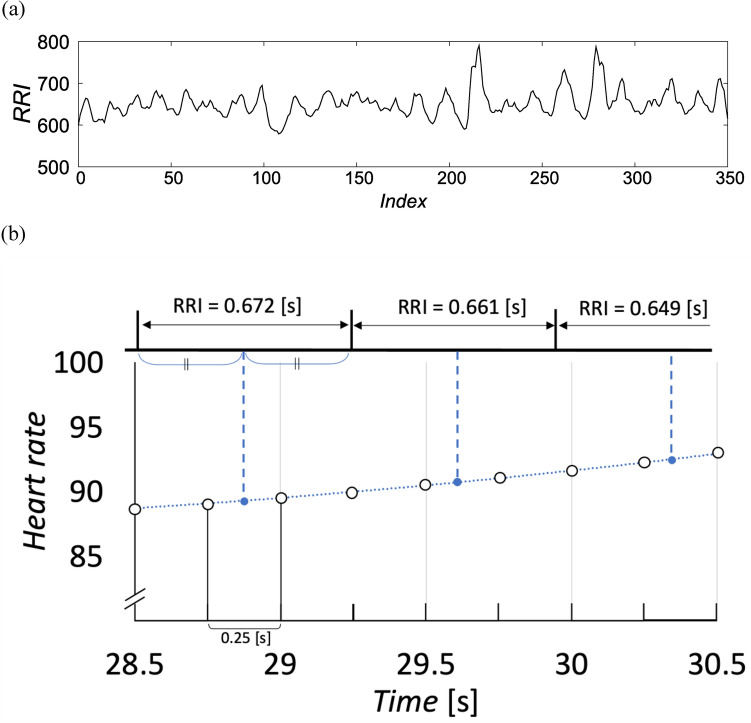
Calculation procedure of instantaneous heart rate. (**a**) Original RRI (Participant B, Trial 1). (**b**) Schematic diagram of the instantaneous heart rate calculation (28.5 [s]—30.5 [s] in the 222 [s] trial). First, the instantaneous heart rate was calculated by the inverse of the RRI and inserted at the midpoint of the RRI (blue points). Then, spline completion (blue dot line) was applied and resampled at 4 [Hz] (white circles).

The instantaneous heart rate was calculated from the measured RR intervals^8^ (Fig. 6). The RR interval was defined as the period between the time one heartbeat, that is, the R wave, was observed and the time the next heartbeat was observed. The midpoints of these two time points were used as temporal locations to insert instantaneous heart rates. The instantaneous heart rate at a given midpoint was calculated as the inverse of the RR interval and inserted at the target midpoint. We interpolated using the *filloutliers* function in MATLAB. Outliers are defined as elements more than three scaled median absolute deviation (MAD) from the median. The outliers were linearly interpolated. Next, the time series of the instantaneous heart rate with constant-time observation was resampled at 4 [Hz] (= 0.25 [s] interval) from the intermittent instantaneous heart rates using the *resample*() function in MATLAB. Therefore, the sample size used to calculate the correlation coefficient was *n* = 889 (222 [s]) for the prepared piece and *n* = 617 (154 [s])–1453 (363 [s]) for the favored piece.

Six (_4_C_2,_ or 2-combinations of 4) pairs of instantaneous heart rate time series were used to calculate the intra-individual correlation coefficients (WSC) for both the prepared and favored pieces of music. To match the number of data used for the BSC calculation with the number of data used for the WSC calculation, instantaneous heart rates were used to calculate the inter-individual correlation coefficients (BSC) obtained from six pairs of four different participants. This calculation, with randomly selected pairs, was repeated 1,000 times to obtain valid results.

The correlation coefficients were integrated to estimate the mean correlation coefficients for each group. A classical meta-analytic method with a fixed effect model^22^ was used to compute the mean correlation coefficients weighted by the degrees of freedom. Fisher’s z-transformation was applied to the raw correlation coefficients. We then obtained the integrated correlation coefficient (*r*) by applying the inverse Fisher’s z-transformation of the arithmetic mean of the correlation coefficients. In this study, the amount of data used to calculate the integrated correlation coefficient corresponded to the length of the time series. Therefore, the sample sizes for the weighted mean were only different for each participant in the case of the favored pieces of music.

To examine nonlinear correlation, phase synchronization ($\varphi$) was calculated based on two recurrence plots of instantaneous heart rates, obtained from the same combination of trials as in the cases of linear correlations. If the influence of a common input is large, the synchronization is almost perfect ($r\approx .90$). In this case, it is sufficient to use Pearson's correlation coefficient. However, if the influence is not large, as considered from previous studies^9,12^, the relationships can be overlooked^23^. Phase synchronization quantifies the degree of coincidence by means of recurrences of time series in a multidimensional state space, embedded in time-delayed coordinates. Phase synchronization becomes high if states recur even in the case that both time series do not show clear first-order correlation.

The phase synchronization $(\varphi )$ is defined^11^ as

$$\varphi = {RR}_{\tau }\left(\varepsilon \right)=\frac{1}{N-\tau }\sum_{i=1}^{N-\tau }\Theta \left(\varepsilon -\Vert {x}_{i}-{x}_{i-\tau }\Vert \right),$$

where $\tau$ is a lag used to calculate the recurrence rate ${RR}_{\tau }$, ${x}_{i} (i=\text{1,2}, \dots , N)$ is the $i$ th state of a time point embedded in the state space, $\varepsilon$ is a threshold, and the $\Theta (\cdot )$ is the Heaviside function.

The parameters of the recurrence plots were determined as follows: (i) the embedding dimension ($m$) was determined using a false nearest neighbor method^11^. To detect the difference between the numbers of false nearest neighbors is sufficiently small, the criterion was set to 0.01 since the differences did not converge exactly on zero in several cases. The $\tau$ was set to a lag that corresponds to the first time point where the slope of mutual information was less than 0.02. The threshold ($\varepsilon$) of the Heaviside function was set so as to the recurrence rate be at 0.1. To calculate phase synchronization, we used MATLAB toolbox of recurrence plot^24^.

Finally, multiple regression analysis was used to determine whether mood predicts synchronization. To examine whether daily fluctuations contribute to synchrony, we chose WSC and WSS, which involved only daily fluctuations without individual differences. The objective variables were WSC and WSS, and the predictor variables were the absolute differences between prior scores of the state emotional subscales for the pairs used for calculating WSC and WSS.

## Data Availability

The data used in this study are available on reasonable request to the corresponding author.

## References

[1] Hata S, Shimokawa T, Arai K, Nakao H (2010). Synchronization of uncoupled oscillators by common gamma impulses: From phase locking to noise-induced synchronization. Phys. Rev. E.

[2] Teramae JN, Tanaka D (2004). Robustness of the noise-induced phase synchronization in a general class of limit cycle oscillators. Phys. Rev. Lett..

[3] Nakao H, Arai K, Kawamura Y (2007). Noise-induced synchronization and clustering in ensembles of uncoupled limit-cycle oscillators. Phys. Rev. Lett..

[4] Schmolke F, Lutz E (2022). Noise-induced quantum synchronization. Phys. Rev. Lett..

[5] Uchida A, McAllister R, Roy R (2004). Consistency of nonlinear system response to complex drive signals. Phys. Rev. Lett..

[6] Mainen ZF, Sejnowski TJ (1995). Reliability of spike timing in neocortical neurons. Science.

[7] Moran PA (1953). The statistical analysis of the Canadian lynx cycle II Synchronization and Meteorology. Aust. J. f Zool..

[8] Pérez P, Madsen J, Banellis L, Türker B, Raimondo F, Perlbarg V, Sitt JD (2021). Conscious processing of narrative stimuli synchronizes heart rate between individuals. Cell Rep..

[9] Madsen J, Parra LC (2022). Cognitive processing of a common stimulus synchronizes brains, hearts, and eyes. PNAS Nexus.

[10] Nomura R, Hino K, Shimazu M, Liang Y, Okada T (2015). Emotionally excited eyeblink-rate variability predicts an experience of transportation into the narrative world. Frontiers in Psychology.

[11] Marwan N, Romano MC, Thiel M, Kurths J (2007). Recurrence plots for the analysis of complex systems. Physics Reports.

[12] Madsen J, Margulis EH, Simchy-Gross R, Parra LC (2019). Music synchronizes brainwaves across listeners with strong effects of repetition, familiarity, and training. Sci. Rep..

[13] Kaneshiro B, Nguyen DT, Norcia AM, Dmochowski JP, Berger J (2020). Natural music evokes correlated EEG responses reflecting temporal structure and beat. NeuroImage.

[14] Léveillé Gauvin H (2018). Drawing listener attention in popular music: Testing five musical features arising from the theory of attention economy. Musicae Scientiae.

[15] Tschacher W, Greenwood S, Ramakrishnan S, Tröndle M, Wald-Fuhrmann M, Seibert C, Meier D (2023). Audience synchronies in live concerts illustrate the embodiment of music experience. Sci. Rep..

[16] Swarbrick D, Bosnyak D, Livingstone SR, Bansal J, Marsh-Rollo S, Woolhouse MH, Trainor LJ (2019). How live music moves us: head movement differences in audiences to live versus recorded music. Frontiers in Psychology.

[17] Ardizzi M, Calbi M, Tavaglione S, Umiltà MA, Gallese V (2020). Audience spontaneous entrainment during the collective enjoyment of live performances: physiological and behavioral measurements. Sci. Rep..

[18] Czepiel A, Fink LK, Fink LT, Wald-Fuhrmann M, Tröndle M, Merrill J (2021). Synchrony in the periphery: inter-subject correlation of physiological responses during live music concerts. Sci. Rep..

[19] Nomura R, Liang Y, Okada T (2015). Interactions among collective spectators facilitate eyeblink synchronization. PLoS One.

[20] Terasaki M, Kishimoto M, Koga A (1992). A construction of a multiple mood scale. Jpn. J. Psychol..

[21] Oode, S., Imai, A., Ando, A. & Taniguchi, T. Evaluation of Kandoh evoked by music: Relation between type of kandoh and affective value of music. *IPSJ Journal*, **50**(3), 1111 –1121 (2009).

[22] Borenstein M, Hedges LV, Higgins JP, Rothstein HR (2011). Introduction to Meta-Analysis.

[23] Sugihara G, May R, Ye H, Hsieh CH, Deyle E, Fogarty M, Munch S (2012). Detecting causality in complex ecosystems. Science.

[24] Marwan, N. Cross Recurrence Plot Toolbox for MATLAB, Version 5.28 (R37), https://tocsy.pik-potsdam.de/CRPtoolbox/, accessed 2023–09–18.

